# A presentation of systemic lupus erythematosus manifesting as abdominal pain: a case report

**DOI:** 10.1186/s13256-025-05451-4

**Published:** 2025-07-31

**Authors:** Chloe Kupelian, DeMarco Bowen, Maria Huang, Begem Lee, Christiane Lenzen, Tiranun Rungvivatjarus

**Affiliations:** 1 Assistant Clinical Professor of Pediatrics, Division of Pediatric Hospital Medicine, University of California San Diego/Rady Children’s Hospital, 3020 Children’s Way, MC 5064, San Diego, CA 92123 USA; 2 Assistant Clinical Professor of Pediatrics, Division of Pediatric Hospital Medicine and Complex Care, University of Wisconsin School of Medicine and Public Health, 600 Highland Avenue, H4/464, Madison, WI 53792 USA

**Keywords:** Systemic lupus erythematosus, Lupus nephritis, Glomerulonephritis, Menstrual cycle

## Abstract

**Background:**

Systemic lupus erythematosus is a multisystem inflammatory disease with a broad range of clinical and serologic presentations. The heterogeneity of presentation poses diagnostic challenges for the clinician, and a high index of suspicion is required. Classification systems exist for both clinical and immunologic criteria; however, they may lack sensitivity in assisting with diagnosis of atypical presentations. We present a case of an initial presentation of systemic lupus erythematosus consisting of nonspecific gastrointestinal symptoms with clinical and immunologic findings that fluctuated with menstrual cycles.

**Case presentation:**

A 15-year-old Hispanic female initially presented with 2 days of epigastric abdominal pain, non-bloody and non-bilious emesis, and diarrhea. There was no fever, rash, weight loss, arthralgias, or dysuria. Menses started 1 day prior to presentation. She was persistently hypertensive throughout her admission. She developed respiratory distress with supplemental oxygen requirement due to pleural effusions identified on chest x-ray. Computed tomography of the abdomen showed large-volume ascites. Extensive evaluation was negative for malignancy and cardiac, gastrointestinal, or infectious etiologies. She demonstrated hypocomplementemia, which self-resolved without intervention. She initially had proteinuria, which resolved after menstruation. She was discharged without a specific diagnosis as her clinical status improved. She presented 2 weeks later for recurrent symptoms at the start of her next menstrual cycle with hypocomplementemia and proteinuria that persisted after menses. Elevated 24-hour urine protein led to a kidney biopsy, which showed mesangial proliferative lupus nephritis class II. The patient was formally diagnosed with systemic lupus erythematosus.

**Conclusion:**

We present a case of new onset systemic lupus erythematosus with initial gastrointestinal symptoms occurring and receding concomitantly with the patient’s menstrual cycle. Interpretation of the urinalysis was complicated by active menses, and both hematuria and proteinuria initially resolved at the completion of her menstrual cycle. In addition, her symptoms and hypocomplementemia resolved without intervention, making the diagnosis more challenging with insufficient clinical criteria for systemic lupus erythematosus. Clinicians should maintain a high index of suspicion for autoimmune disorders, as symptoms may unfold over time. Although rare, systemic lupus erythematosus may initially present with gastrointestinal symptoms without other classic clinical findings. Absence of serologic criteria and spontaneous resolution of hypocomplementemia also add to the novelty of this case.

## Background

Systemic lupus erythematosus (SLE) is an autoantibody-mediated disease that can affect any of the major organ systems but typically involves the hematologic, musculoskeletal, mucocutaneous, and renal systems. The American College of Rheumatology (ACR) first developed classification criteria in 1982 and revised these in 1997 to enhance sensitivity and specificity for diagnosis [1]. Given further advancements in understanding of the disease, the criteria were refined in 2012 by the Systemic Lupus International Collaborating Clinics (SLICC) to include requirement of four features (including at least one immunologic feature) or biopsy-proven lupus nephritis with a positive antinuclear antibody (ANA) or anti-double-stranded deoxyribonucleic acid (anti-DS DNA) titer. While this increased sensitivity for diagnosis, it also reduced specificity compared with the ACR criteria [2]. In 2019, the European Alliance of Associations for Rheumatology/American College of Rheumatology (EULAR/ACR) sought to improve reliability of diagnosis by defining positive ANA as an entry criterion, which improved both sensitivity and specificity for diagnosis in adult-onset SLE compared with the two prior classification systems [1]. Recent studies have suggested that SLICC-2012 criteria are more sensitive in diagnosing pediatric versus adult-onset SLE when compared with ACR-1997 and EULAR/ACR-2019 [3, 4]. While hypocomplementemia is a diagnostic criterion for both SLICC-2012 and EULAR/ACR-2019 classification systems, C3 and C4 are not always associated with disease flares [5].

Approximately 90% of people with SLE are female, and 40% will develop lupus nephritis [6]. Early detection of the disease is essential to reduce organ damage and prevent exacerbations. The clinical presentation of SLE varies widely, and although classification systems exist, diagnostic challenges exist in the early stages, and diagnosis is often delayed [7]. In this case report, we present an adolescent female with recurrent nonspecific gastrointestinal (GI) symptoms as the first manifestation of SLE.

## Case presentation

A 15-year-old previously healthy Hispanic female presented to the emergency department (ED) with 2 days of epigastric abdominal pain, non-bloody (NB) and non-bilious emesis, and NB diarrhea. She endorsed abdominal bloating and early satiety. There was no report of sick contacts or recent travel. She was seen 1 month prior for similar symptoms and clinically diagnosed with gastritis. At that time, her symptoms self-resolved without intervention or identified etiology. Review of systems was negative for fever, rashes, unintentional weight loss, arthralgias, and dysuria. Her menstrual period started 1 day prior to presentation, with report of regular menstruation. Family history was negative for rheumatologic, gastrointestinal, or malignant conditions.

Physical exam was notable for a heart rate of 120 beats per minute and a blood pressure (BP) of 133/96 mm Hg. Body mass index (BMI) was in the 86th percentile. No prior BP measurements were available for review, although the patient denied a prior history of hypertension. She was non-toxic in appearance. Cardiac exam showed no murmur or rubs. Breath sounds were clear bilaterally. Abdominal exam was positive for distention and epigastric tenderness with palpation, with no guarding or rigidity. Exam was negative for hepatosplenomegaly, costovertebral angle tenderness, peripheral edema, rashes, or oral ulcerations.

Initial labs showed normal complete blood cell count, electrolytes, kidney function, liver enzymes, lipase, and inflammatory markers. Urine pregnancy and drug screen were both negative. Urinalysis dipstick showed 2+ ketones, 3+ blood, 2+ protein, and negative nitrite and leukocyte esterase, while on her menses. Pelvic ultrasound (US) with Doppler was negative for ovarian torsion. Computed tomography of the abdomen/pelvis showed diffuse severe enterocolitis with large volume ascites. Infectious workup was all negative, including urine culture, *H. pylori* stool antigen, GI pathogen panel, ova and parasites, stool culture, and *Clostridioides difficile* toxin.

**Fig. 1:**
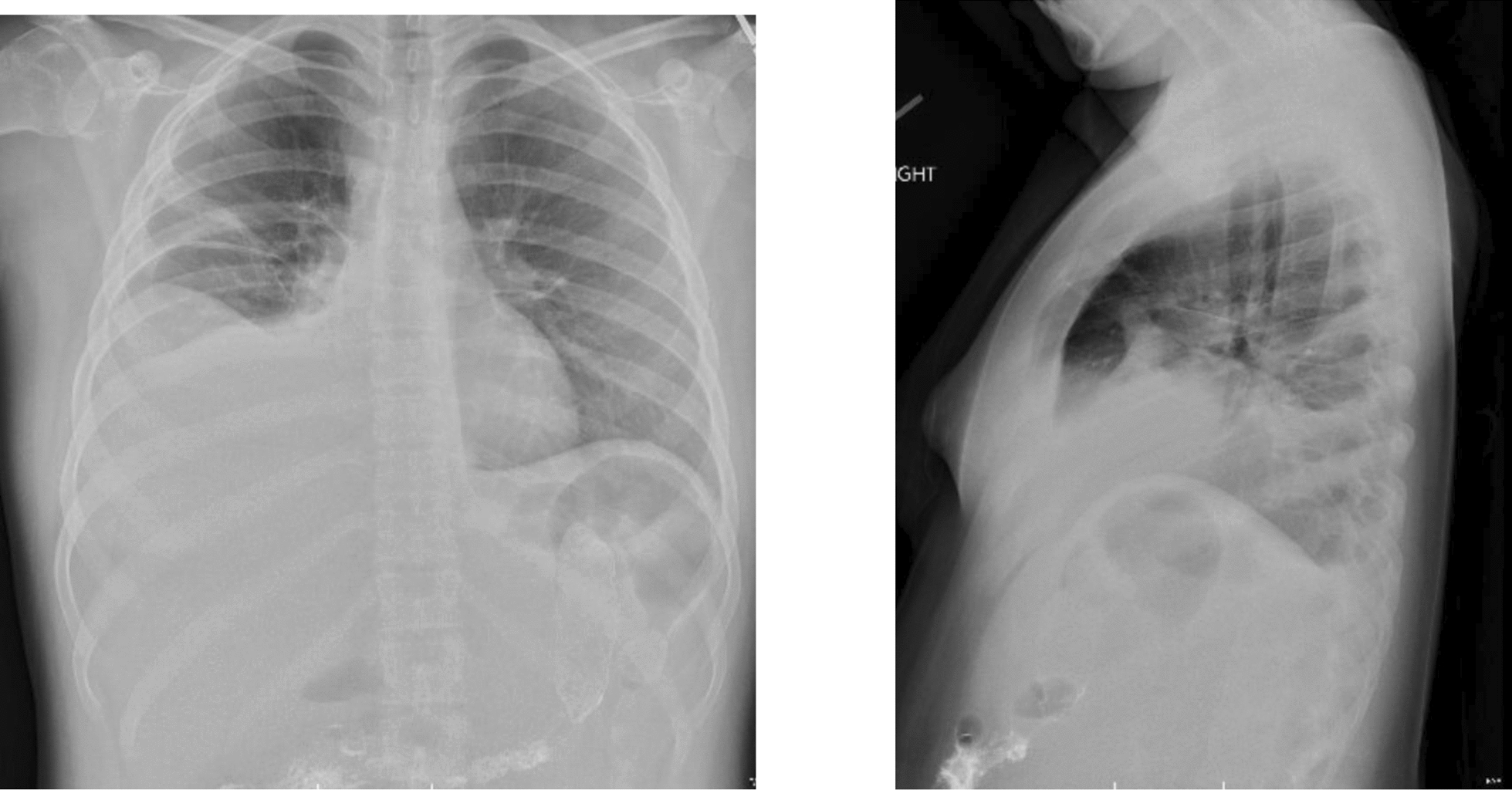
Two-view chest radiograph depicting bilateral pleural effusions (right greater than left) during patient’s first hospitalization

Following admission, her symptoms progressed to dyspnea and worsening abdominal distention. She required supplemental oxygen and scheduled diuresis for therapeutic relief. Chest radiograph revealed bilateral moderate-sized pleural effusions (Fig. 1). Echocardiogram showed normal function without pericardial effusion. Tumor lysis labs and peripheral smear were both normal. Repeat lab evaluation was significant for hypoalbuminemia of 2.4 g/dL. Given abdominal distention in the setting of hypoalbuminemia, evaluation of the etiologies of protein-losing enteropathy was initiated. Fecal calprotectin was mildly elevated at 263 mg/kg. Celiac screen and alpha-1-antitrypsin stool testing were both negative. Gastroenterology team was consulted, and she underwent endoscopy and colonoscopy, which showed normal mucosa and biopsies, ruling out inflammatory bowel disease.

She was noted to be hypertensive throughout her hospitalization, with systolic BP ranging between 130 mm Hg and 140 mm Hg. Plasma renin, aldosterone levels, thyroid function test, and renal ultrasound with Doppler were all normal. Echocardiogram was negative for coarctation of the aorta or left ventricular hypertrophy. Repeat urinalysis was collected after completion of her menses, which was normal and showed resolution of prior hematuria and proteinuria.

Given no clear source for her presentation, rheumatologic etiologies were considered. Complement levels C3 and C4 were collected and were both decreased at 46 mg/dL (75–175 mg/dL) and 8 mg/dL (22–45 mg/dL), respectively, initially suggestive of SLE as a diagnosis; however, C3 normalized to 76 mg/dL prior to discharge without intervention, arguing against SLE. ANA titer was elevated (1:80). Additional antibody titers, including anti-double stranded DNA (DS DNA), anti-neutrophil cytoplasmic antibody, anti-Smith, anti-ribonucleotide protein, antiphospholipid, beta-2-glycoprotein, cardiolipin, anti-streptolysin O, anti-Ro, and anti-La, were all collected and negative. Rheumatology was consulted and had low suspicion for autoimmune disease, given normalization of complement levels without intervention and absence of classic clinical findings for a specific rheumatologic disorder. The patient gradually improved throughout her hospital stay and was weaned off oxygen and diuretic therapy. She was discharged home on hospital day 9 with plans for outpatient follow-up.

Subsequently, 2 weeks later, she returned to the ED with recurrence of epigastric abdominal pain, vomiting, and diarrhea. She was again noted to be on her menses. Her labs on initial presentation showed normal electrolytes, kidney function, albumin, aspartate and alanine transaminases, and inflammatory markers. She had new thrombocytopenia with platelet count of 74 TH/uL (150–400 TH/uL) with normal coagulation panel, arguing against a consumptive process and suggestive of possible immune-mediated destruction or bone marrow suppression. She was readmitted to the hospital. The following day, her platelet count normalized without intervention.

**Fig. 2:**
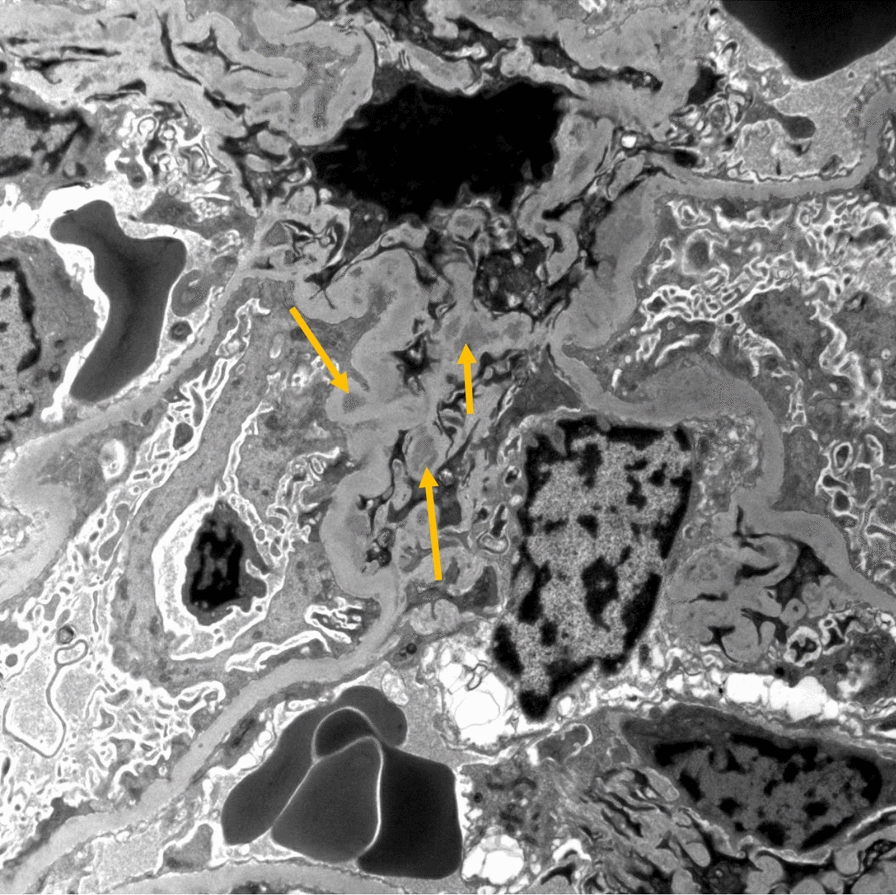
Electron microscopy, showing electron-dense mesangial deposits (yellow arrows), consistent with lupus nephritis class II

**Fig. 3:**
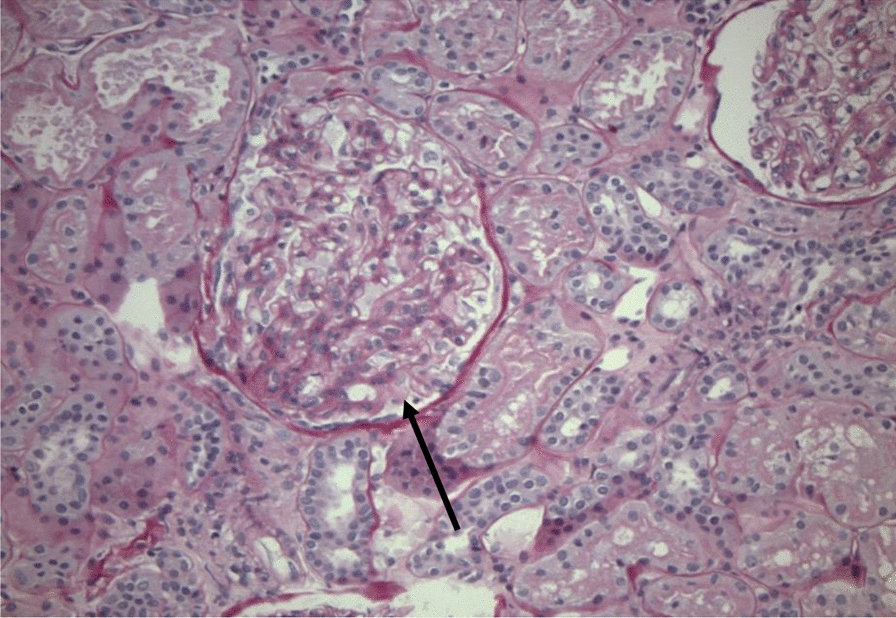
Light microscopy, with a glomerulus showing mesangial hypercellularity and proliferation (black arrow), consistent with lupus nephritis class II

Complement levels were repeated, and C4 remained low at 8 mg/dL and previously normal C3 at time of discharge now decreased to 49 mg/dL. In light of hypocomplementemia, antibody titers from the prior admission, including anti-DS-DNA and anti-Smith, were repeated and all remained negative. She continued with elevated blood pressures and required scheduled antihypertensive medication. Kidney function remained normal. A 24-hour urine collection was obtained after completion of her menses and revealed mildly elevated 24-hour protein of 365 mg (reference range of 100–150 mg/24 hours). Secondary to these findings and lack of a clear diagnosis, a kidney biopsy was obtained and revealed mesangial proliferative lupus nephritis (LN) class II, with electron microscopy showing mesangial deposits with diffuse podocyte effacement (Figs. 2 and 3). The patient received the formal diagnosis of SLE. She met SLICC criteria on the basis of the presence of lupus nephritis in the setting of positive ANA titer. She received intravenous pulse steroids with methylprednisolone 1000 mg/day for 3 days total. She also began therapy with hydroxychloroquine 200 mg daily and mycophenolate 500 mg twice daily with subsequent improvement in her clinical status. She was discharged home with the above mentioned immunosuppressants along with 60 mg of prednisone daily for 1 month until her scheduled follow-ups with rheumatology and nephrology. On follow-up, 1 month later, her platelet count, C3, and C4 all normalized, and proteinuria resolved with appropriate medication compliance. She had resolution of symptoms without recurrence of vomiting or abdominal pain. Given clinical and immunologic improvement, her steroids were gradually tapered over the next several weeks.

## Discussion

We present a case of new onset SLE with class II LN confirmed with kidney biopsy and electron microscopy. Patients with class II LN typically present with preserved kidney function and do not require additional immunosuppression beyond standard SLE therapy. In cases such that of our patient with either nephrotic-range proteinuria or evidence of lupus podocytopathy (defined as diffuse podocyte effacement on electron microscopy), glucocorticoid therapy is recommended with consideration of additional immunosuppressive agents to induce remission and prevent relapse. [7]

Diagnosis of SLE was delayed owing to initial symptoms consisting of GI manifestations without hematologic, cutaneous, or synovial involvement. Although less common, SLE may initially present with nonspecific GI symptoms, including nausea, vomiting, and abdominal pain, leading to potential for delayed diagnosis [8–10]. Evidence of serositis with pleural effusions and large-volume ascites developed later during the initial hospitalization, which interestingly improved at the completion of the patient’s menstrual cycle. Additional diagnostic challenges included negative serologic titers. Our patient failed to meet both SLICC-2012 and EULAR/ACR-2019 criteria for the diagnosis of SLE during her first admission. Her ANA titers were initially elevated; however, a positive ANA is not specific for SLE and has been shown to be elevated even in healthy individuals without known rheumatologic conditions [11, 12]. Autoantibody titers specific for SLE (anti-DS DNA and anti-Smith), which were monitored across two different menstrual cycles, were negative during both hospitalizations, delaying her diagnosis in the absence of classic clinical criteria. Although specific for the diagnosis, the titers may have poor predictive value early in the disease. [13, 14]

Furthermore, our patient developed hypocomplementemia and isolated thrombocytopenia, both of which resolved without intervention. Spontaneous resolution of hypocomplementemia and thrombocytopenia is an atypical presentation of SLE and adds to the novelty of our patient’s presentation. Prior studies have suggested that serum C3 or C4 measurements may have poor clinical utility in identifying an SLE flare when used in isolation without other diagnostic markers. [15]

While she did have proteinuria, this was in the setting of active menses, complicating interpretation of the urinalysis and ability to finalize a diagnosis. Proteinuria subsequently resolved after completion of her menses. It was not until her second admission where she had an elevated 24-hour protein concentration that prompted the kidney biopsy, which confirmed the diagnosis.

Her diagnosis was further delayed by resolution of clinical symptoms and hypocomplementemia at the end of her menses. Interestingly, the patient presented both times at the start of menstruation. Previous studies have suggested that hormonal fluctuations during the menstrual cycle may exacerbate clinical features of SLE [16]. Menstrual-associated lupus flares are thought to be driven by cyclical hormonal fluctuations. Estrogen, which promotes B cell activation and type I interferon expression and delays clearance of apoptotic cells, contributes to proinflammatory immune activity. However, progesterone exerts immunomodulatory effects by enhancing regulatory T cell function and suppressing Th1 and Th17 responses. During the late luteal phase and menstruation, both estrogen and progesterone levels decline. The withdrawal of progesterone’s immunosuppressive influence, estrogen fluctuations, and increased levels of proinflammatory cytokines such as IL-6 and TNF-α, may play a role in triggering disease activity in females with SLE [16, 17].

While there was symptom recurrence at the start of her menses during both hospitalizations, we are unable to definitively link the patient’s menstrual cycle with her SLE flare owing to the absence of endometrial immunopathology.

## Conclusion

We present a case of new-onset SLE, with initial symptoms occurring concomitantly with the patient’s menstrual cycle. Diagnosis was delayed owing to detection of proteinuria, which was presumed to be due to active menstruation, complicating the diagnosis. Furthermore, her initial presentation began with recurrent GI symptoms without hematologic, cutaneous, or synovial involvement. This was further complicated by spontaneous improvement of hypocomplementemia and insufficient criteria for diagnosis of SLE. Clinicians should maintain a high index of suspicion for autoimmune disorders; symptoms may unfold over time and even fluctuate with normal hormonal changes.

## Data Availability

Not applicable.

## Abbreviations

SLE: Systemic lupus erythematosus
ACR: The American College of Rheumatology
SLICC: Systemic Lupus International Collaborating Clinics
EULAR/ACR: European Alliance of Associations for Rheumatology/American College of Rheumatology
ANA: Antinuclear antibody
Anti-DS DNA: Anti-double-stranded deoxyribonucleic acid
GI: Gastrointestinal
NB: Non-bloody
ED: Emergency department
BP: Blood pressure
LN: Lupus nephritis
